# Evaluation and Verification of Channel Transmission Characteristics of Human Body for Optimizing Data Transmission Rate in Electrostatic-Coupling Intra Body Communication System: A Comparative Analysis

**DOI:** 10.1371/journal.pone.0148964

**Published:** 2016-02-11

**Authors:** Yuhwai Tseng, Chauchin Su, Yingchieh Ho

**Affiliations:** 1 Department of Electrical and Control Engineering, National ChiaoTung University, Hsin-Chu City, Taiwan; 2 Electrical Engineering Department, National Dong Hwa University, Hualien, Taiwan; Chongqing University, CHINA

## Abstract

**Background:**

Intra-body communication is a new wireless scheme for transmitting signals through the human body. Understanding the transmission characteristics of the human body is therefore becoming increasingly important. Electrostatic-coupling intra-body communication system in a ground-free situation that integrate electronic products that are discretely located on individuals, such as mobile phones, PDAs, wearable computers, and biomedical sensors, are of particular interest.

**Materials and Methods:**

The human body is modeled as a simplified Resistor-Capacitor network. A virtual ground between the transmitter and receiver in the system is represented by a resister-capacitor network. Value of its resistance and capacitance are determined from a system perspective. The system is characterized by using a mathematical unit step function in digital baseband transmission scheme with and without Manchester code. As a result, the signal-to-noise and to-intersymbol-interference ratios are improved by manipulating the load resistor. The data transmission rate of the system is optimized. A battery-powered transmitter and receiver are developed to validate the proposal.

**Results:**

A ground-free system fade signal energy especially for a low-frequency signal limited system transmission rate. The system transmission rate is maximized by simply manipulating the load resistor. Experimental results demonstrate that for a load resistance of 10k−50k Ω, the high-pass 3 dB frequency of the band-pass channel is 400kHz−2MHz in the worst-case scenario. The system allows a Manchester-coded baseband signal to be transmitted at speeds of up to 20M bit per second with signal-to-noise and signal-to-intersymbol-interference ratio of more than 10 dB.

**Conclusion:**

The human body can function as a high speed transmission medium with a data transmission rate of 20Mbps in an electrostatic-coupling intra-body communication system. Therefore, a wideband signal can be transmitted directly through the human body with a good signal-to-noise quality of 10 dB if the high-pass 3 dB frequency is suitably selected.

## Introduction

A human body comprises such conductive materials as blood, living tissue, and extracellular and intracellular fluids, [1–16], which can serve as transmission media. *Intra-body communication* (IBC) involves using such media; linking of these media to discrete electronic devices such as mobile phones, PDAs, wearable computer, biomedical sensors and actuators that are attached to the human body to monitor instantaneous human health status and the surrounding environment, has recently been considered [17–32].

IBC systems are categorized as electromagnetic waveguide (EMW) and electrostatic coupling (ESC) systems. An EMW system generates electromagnetic waves using both positive and negative terminals of transmitter and receiver with an electrode, and treats the human body as a waveguide for signal transmission. The impedance of the body between transmitter and receiver in EMW system is a complex resistor-capacitor (*RC*) network [24–27]. *RC*-based body impedances reduce the channel bandwidth, weaken the signal energy, especially at high frequency, and finally limit the data transmission rate below 100k bit per second (bps).

In an ESC IBC system, the positive terminal of the transmitter and the receiver are connected to the human body using an electrode. Negative terminals are opened to keep the system ground-free. The ground of the transmitter and the receiver are at different potentials. The environment provides signal return path. The path on the system generates a band-pass channel with high-pass and low-pass 3dB cutoff frequencies, which vary as function of the environment, the electrical properties of the human body, and the load resistance. The channel degrades the quality of the signal and reduces the signal-to-noise (*SNR*) and the signal-to-intersymbol-interference ratio (*SIR*) of the system. The channel limits the transmission rate of the developed system below 2M bit per second (BPS) [20–27]. The measurement methods [28–32] that are currently used to measure the transmission characteristic of the channel are shortcoming, in that all corresponding measurement instruments share a common ground of the power line. The ground provides a signal return path between the transmitter and the receiver. Such a signal return path is not the same as that of the ESC IBC channel. Thus, a certain model mapping must be carried out by transforming the channel model using a metal wire as the ground return loop to the one that uses the environment as the ground return loop.

This work proposes an ESC IBC band-pass system that is based on a baseband transmission scheme and uses an equivalent *RC* circuit model with a signal return path that is modeled as capacitors. The parameters of the system are evaluated on a system perspective that was explained elsewhere [7]. Based on the de-convolution of a square test waveform, the frequency response of a bandpass system that is based on an ESC IBC channel is obtained and procedure for measuring body impedance is simplified. The load resistor and square test waveform are selected such that the bandpass system can be translated into a high- or low-pass system; the body impedances can then be evaluated straightforwardly.

A comparative analysis that uses the unit step function is conducted to obtain the channel impulse response for two digital baseband transmission schemes- with and without Manchester code. The load resistor, *R*_*L*_, can be chosen to maximize simultaneously the data transmission rate, the *SNR*, and the *SIR*. A ground-free system with a battery-powered transmitter and receiver are developed to validate the proposal. The remainder of this paper is organized as follows. Section 2 describes materials and the methodology. Section 3 determines the *SNR* and *SIR* based on the channel model and data transmission pattern, such that the optimal compromise among the *SNR*, *SIR* and data transmission rate can be achieved. Section 4 presents a battery-powered transmitter and receiver with a different ground to verify the proposed methodology. Finally, Section 5 draws the conclusions.

## Materials and Methods

### Model of a signal return path in an ESC IBC system

**Fig 1:**
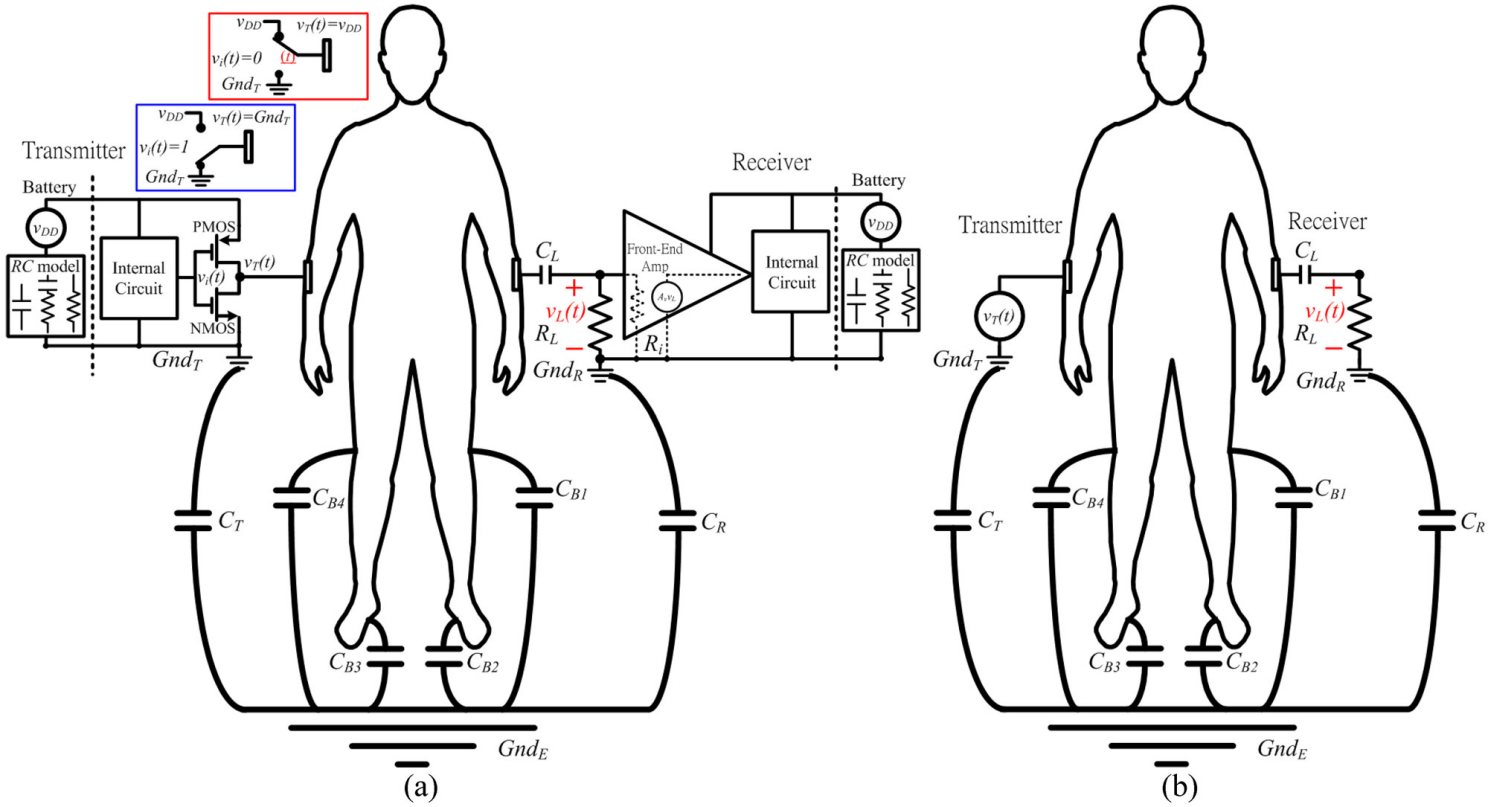
Model of a signal return path in an ESC IBC system. (a) A complete circuit model and (b) a simplified circuit model of a transmitter and a receiver with different battery-powered sources.

The transmission characteristics of an ESC IBC system for high-speed transmission are analyzed. Table 1 presents the nomenclature that is used in describing the system that is developed in this study. Fig 1a displays a circuit model of a transmitter and a receiver with different battery-powered sources, which is currently used in the system. The positive terminals of the transmitter and the receiver are connected to the human body using an electrode. Negative terminals are opened to keep the system ground-free. *Gnd*_*T*_ and *Gnd*_*R*_ represent the grounds of the transmitter and the receiver, respectively. Since *Gnd*_*T*_ ≠ *Gnd*_*R*_, a signal return path from the *Gnd*_*T*_ and *Gnd*_*R*_ through the environment to the earth ground are modeled as capacitors *C*_*T*_ and *C*_*R*_, respectively.

**Table 1:** Nomenclature that is used in describing the system.

*A*	Amplitude of the transmitted digital signal	*A*_*v*_	Gain of the front-end amplifier
*C*_*B*_,*C*_*B1*_~*C*_*B4*_	Capacitance from the earth ground to the human body	*C*_*b*_	Membrane capacitance of red blood cells
*C*_*L*_	Capacitor before the front-end amplifier equal to 100nF	*C*_*m*_	Membrane capacitance of muscle tissues
*C*_*n*_	Capacitance from the power line to the human body	*C*_*R*_	Capacitance from the earth ground to the transmitter
*C*_*T*_	Capacitance from the earth ground to the transmitter	*C*_*t*_	Tissue capacitance of the body parts
*C*_*X*_	Skin capacitor	*G*	Gain of the ESC IBC system presented by unit step function = $\frac{A}{Z_{B}C_{B}2\omega_{n}\zeta \sqrt{1-\frac{1}{\zeta^{2}}}}$
*G*_*f*_	Gain factor of the ESC IBC system = $\frac{1}{Z_{B}C_{B}}$	*GND*_*E*_	The earth ground
*GND*_*R*_	The receiver ground	*GND*_*T*_	The transmitter ground
*H(s)*	Transfer function of the ESC IBC system in s-domain	*R*_*b*_	Intracellular fluid resistance of red blood cells
*R*_*e*_	Extracellular fluid resistance	*R*_*i*_	Input impedance of the front-end amplifier
*R*_*L*_	Load resistor of the receiver	*R*_*m*_	Intracellular fluid resistance of muscle tissues
*R*_*X*_	Skin resistance	*V*_*L*_*(s)*	Received voltage across load resistor in s-domain
*V*_*T*_*(s)*	Transmitter output voltage in s-domain	*T*	Data duration
*Z*_*B*_	Over all body impedance $R_{e}//\left(R_{m}+\left|\frac{1}{sC_{m}}\right|\right)//\left(R_{b}+\left|\frac{1}{sC_{b}}\right|\right)//\left|\frac{1}{sC_{t}}\right|$	*f*_*b*_	Data frequency
*f*_*h*_	High-pass 3dB cut off frequency	*f*_*l*_	Low-pass 3dB cut off frequency
*i*_*dn*_	Displacement current flows from the power line to the human body	*n*_*x*_	The number of bits of transmitted data at the *x*^*th*^ transition
*v(t)*	Random digital signal	*v*_*DD*_	Output voltage of the battery
*${\tilde{v}}_{LR}$*	The mean amplitude of *v*_*L_Sq*_*(t)*	*v*_*L_Sq*_*(t)*	The square waveform with a duty cycle of 50%, *n*_*x*_ = *m* and *(m-1)T*<*t*≦*mT* received at the load resistor
*v*_*L*_*(t)*	Received voltage across the load resistor in time-domain	*v*_*nm*_	Noise margin
*v*_*n*_	The power line noise of the human body	*v*_*nl*_	The power line noise across the load resistor
*v*_*T*_*(t)*	The transmitter output voltage in time-domain	*v*_*t*_*(t)*	Input voltage of the CMOS output buffer in time-domain
*v*_*x*_*(t)*	The transmitted voltage at the electrode of the receiver in time-domain	*x*	The number of data transitioned from 1 to 0 or 0 to 1
*ω*_*n*_	Natural frequency	*ς*	Damping factor

The battery is modeled as a voltage source *v*_*DD*_ in series with a *RC* network [33] that can be categorized into first-order *RC* [34–36], second-order *RC* [37–39], and third-order *RC* network [40]. The effect of these *RC* networks on the battery can be neglected since each resistance is sufficiently small below several tens mΩ and each capacitances is larger than several tens of Farads. The battery model can be simplified as a voltage source.

The transmitter consists of a battery, an internal circuit and an output buffer. The buffer is a CMOS inverter that is composed of a PMOS and a NMOS transistor. *v*_*t*_(*t*) and *v*_*T*_(*t*) represent the output voltage of the internal circuits and the buffer, respectively. When *v*_*t*_(*t*) is low, PMOS is on and NMOS is off, the *v*_*T*_(*t*) connects to the positive terminal of the battery, at a voltage of *v*_*DD*_ (shown as a block outlined in red in Fig 1a). When *v*_*t*_(*t*) is high, PMOS is off and NMOS is on, *v*_*T*_(*t*) is connects to the ground of the transmitter *Gnd*_*T*_ (and is shown as a block outlined in blue in Fig 1a). The operation of the transmitter output port is dominated by the buffer output port. Therefore, the output of the transmitter can be represented by the buffer output *v*_*T*_*(t)*.

The receiver consists of a capacitor *C*_*L*_, a load resistor *R*_*L*_, battery, an internal circuit and a front-end amplifier. *C*_*L*_ isolates the DC signal that enters the human body. The load resistor *R*_*L*_ before the receiver is connected in series to the ground of the receiver *Gnd*_*R*_. The amplifier is modeled as a small-signal equivalent circuit. *A*_*v*_ denotes gain of the amplifier. *R*_*i*_ is the input impedance of the amplifier in parallel with the *R*_*L*_. The contribution of *R*_*i*_ can be neglected because it exceeds several hundreds of MΩ, and so is much larger than the *R*_*L*_. The received signal and noise across the *R*_*L*_ critically affects the system performance, which can be optimized simply by analyzing the role of *R*_*L*_ in the ESC IBC system. Based on the above description, Fig 1a is simplified as Fig 1b.

### Circuit Model of the Band-Limited-ESC IBC Channel

A previous study demonstrated that living human body tissues contain no inductive components. The electrical properties of living tissues can be modeled using a resister and capacitor. Traditionally, biomedical engineer have viewed the human body as a resistor network [41–43]. Owing to the extremely low frequency biopotential signal that is generated from the body, as revealed by ECG, EEG, and EMG, the reactance of the capacitance of living tissues in the body is larger than that of the network of resistive components, which are regarded as an open circuit.

**Fig 2:**
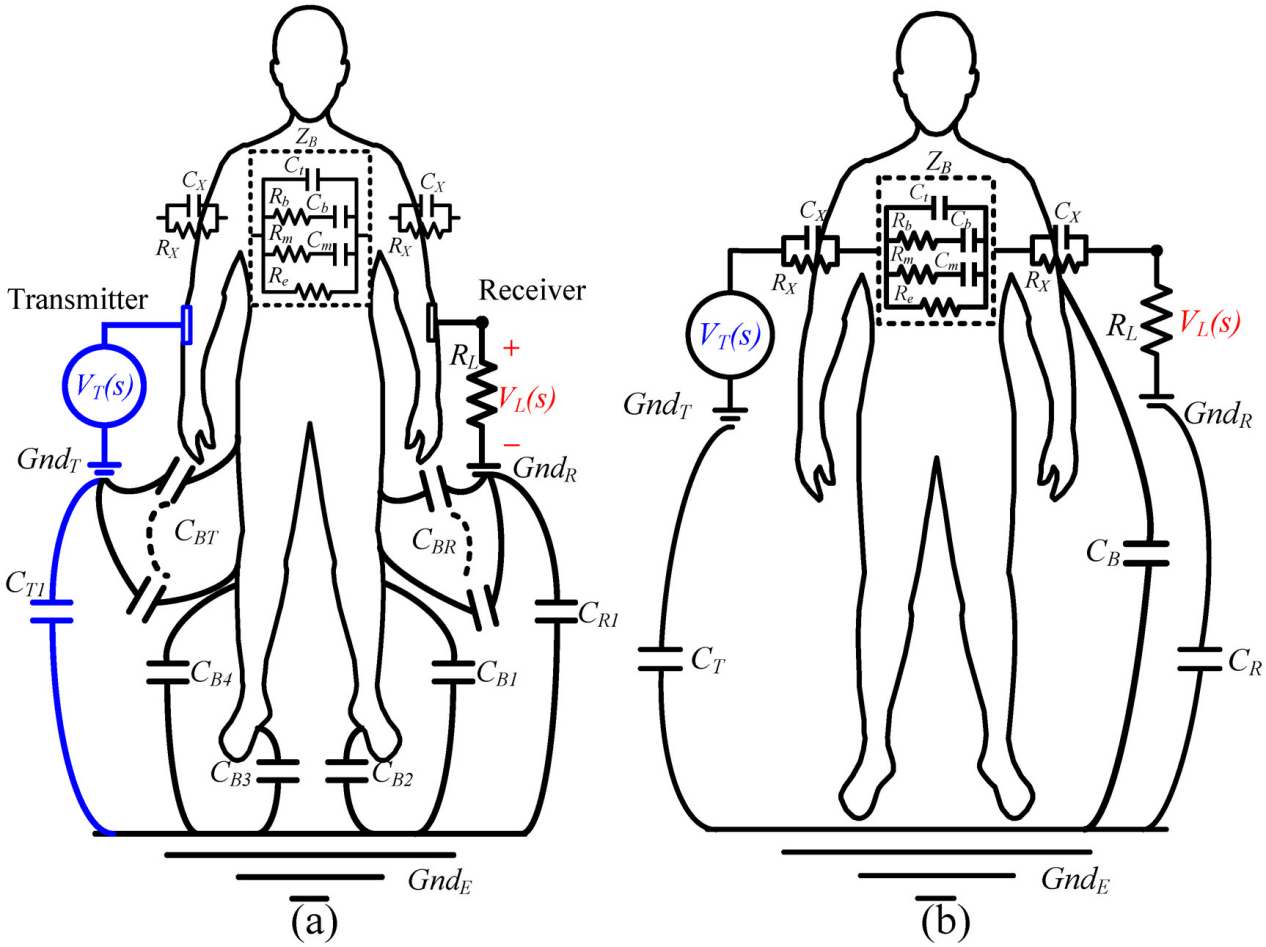
The model of an ESC IBC syatem, (a) the *RC* circuit model, (b) the simplified circuit model.

Communication engineers model the human body as a network of capacitors [17–18] because the mean body impedance is smaller than the paralleled resistances of body tissue when high-frequency data, with a frequency of over 500k Hz, are being transmitted through it. Since low- and high- frequency broadband signal are transmission in the human body, the resistance and capacitance of the body should be considered in designing a digital baseband ESC IBC system. Fig 2a displays a circuit model of an ESC IBC channel in which the human body is simplified as a multi-time-constant circuit [1–5]. The earth functions a virtual gorund (*Gnd*_*E*_) between the transmitter and the receiver ground (*Gnd*_*T*_ and *Gnd*_*R*_). The environment around the system, including the earth, human body, the atmosphere, and so on, forms a signal return path between the transmitter and the receiver ground. One signal return path that comprises the capacitors: *C*_*BR*_, *C*_*BT*_, *C*_*B1*_~*C*_*B4*_, and *C*_*T1*_ and the body impedance can be modeled as $\left|\frac{1}{sC_{BR}}\right|+\left[\left|\frac{1}{sC_{B1}}\right|//\left[2\left(\left|\frac{1}{sC_{X}}\right|//R_{X}\right)+Z_{B}+\left(\left|\frac{1}{sC_{BT}}\right|//\left(\left|\frac{1}{sC_{T1}}\right|+\left|\frac{1}{sC_{B4}}\right|\right)\right)\right]\right]$. Since the capacitance *C*_*B1*_ exceeds several hundred pico-Farads, and so exceeds the capacitances *C*_*BT*_ and *C*_*T1*_, which are less than several pico-Farads, $\left|\frac{1}{sC_{B1}}\right|$ is less than $\left[2\left(\left|\frac{1}{sC_{X}}\right|//R_{X}\right)+Z_{B}+\left(\left|\frac{1}{sC_{BT}}\right|//\left(\left|\frac{1}{sC_{T1}}\right|+\left|\frac{1}{sC_{B4}}\right|\right)\right)\right]$ that it can be neglected for a parallel capacitor circuit [7, 17–19]. The signal return path can be regarded as a series-impedance circuit of the capacitors *C*_*BR*_, and *C*_*B1*_ and body impedance. Fig 2b is simplified version of Fig 2a. The signal return path between the transmitter, the receiver and the earth is simply modeled as capacitors *C*_*T*_ and *C*_*R*_. The *C*_*T*_ and *C*_*R*_ are the simplified parallel capacitor circuit with capacitance of $\left|\frac{1}{sC_{T1}}\right|//\left(\left|\frac{1}{sC_{BT}}\right|+\left|\frac{1}{sC_{B4}}\right|\right)$ and $\left|\frac{1}{sC_{R1}}\right|//\left(\left|\frac{1}{sC_{BR}}\right|+\left|\frac{1}{sC_{B1}}\right|\right)$, respectively.

Here, *C*_*R*_ = *C*_*T*_ is assumed. The typical resistance, *R*_*X*_, and capacitance, *C*_*X*_, of the dry skin are of the order of several hundreds of kΩ and several tens of nF, respectively. Since this work concerns data transmission at rate greater than 500k bps, *R*_*X*_ in parallel with *C*_*X*_ is neglected. The impedance of the skin is neglected since the reactance $\left|\frac{1}{sC_{X}}\right|$ is about zero—less than the reactance $\left|Z_{B}\right|$, $\left|\frac{1}{sC_{T}}\right|$ and *R*_*L*_ with which is in parallel, as presented in Fig 2. Accordingly, system transfer functions *H(s)* represented in s-domain is derived as,

$$H(s)=\frac{V_{L}(s)}{V_{T}(s)}=G_{f}\times \frac{s}{s^{2}+2\varsigma \omega_{n}s+\omega_{n}^{2}},$$

$$\left[\begin{array}{c} G_{f}\\ \omega_{n}^{2}\\ 2\varsigma \omega_{n} \end{array}\right]=\left[\begin{array}{cccc} 1 & 0 & 0 & 0\\ \frac{1}{R_{L}C_{T}} & \frac{1}{R_{L}C_{B}} & 0 & \frac{1}{Z_{B}C_{T}}\\ 1 & 1 & 1 & 1 \end{array}\right]\left[\begin{array}{c} \frac{1}{Z_{B}C_{B}}\\ \frac{1}{Z_{B}C_{T}}\\ \frac{1}{R_{L}C_{B}}\\ \frac{1}{R_{L}C_{T}} \end{array}\right].$$ (1)

where *G*_*f*_ is a gain factor; *ς* is a damping factor, and *ω*_*n*_ is the natural frequency of the system. Eq 1 shows that the system is a bandpass channel with lower and upper 3dB cutoff frequencies (*f*_*h*_, *f*_*l*_):

$$f_{h}=\frac{\omega_{n}}{2\pi }\varsigma \left(1-\sqrt{1-\frac{1}{\varsigma^{2}}}\right),\begin{array}{cc} & f_{l}=\frac{\omega_{n}}{2\pi }\varsigma \left(1+\sqrt{1-\frac{1}{\varsigma^{2}}}\right) \end{array}.$$ (2)

Eqs 1 and 2 indicate that *f*_*h*_ and *f*_*l*_ are controlled by *Z*_*B*_, *C*_*B*_, *C*_*T*_ and *R*_*L*_. Notably, *f*_*h*_ is independent of *Z*_*B*_ and inversely proportional to *R*_*L*_, and *f*_*l*_ is independent of *R*_*L*_ and inversely proportional to *Z*_*B*_. Since *Z*_*B*_, *C*_*T*_, and *C*_*B*_ are uncontrollable, one can manipulate *R*_*L*_ to change *f*_*h*_.

### Evaluating components

The bioelectric impedances of the body and skin are normally determined separately to facilitate a discussion of their features [8–13]. This work considers a simplified human body and uses its equivalent circuit model in Fig 2 with equal skin and body impedances. Various load resistances can transform a human body into a high-pass or low-pass system. The test stimulus herein is a square waveform. De-convolution is performed to obtain the response of the frequency, amplitude, and phase of a human body. The body impedances are estimated in a specific frequency band. A simplified procedure and a complex system in the s and frequency domain are described as follows.

1. A capacitance *C* in the s domain, $\frac{1}{sC}$, can be translated into the frequency domain $\frac{1}{j2\pi fC}$. The symbol || denotes the magnitude of a complex number. The magnitude of $\frac{1}{sC}$ represents the reactive impedance of *C*, which equals to $\left|\frac{1}{sC}\right|=\left|\frac{1}{j2\pi fC}\right|=\frac{1}{2\pi fC}$.
2. The magnitude of a series *RC* circuit $\left(R+\frac{1}{sC}\right)$ is derived as

$$\left|R+\frac{1}{sC}\right|=\left|R+\frac{1}{j2\pi fC}\right|=\sqrt{R^{2}+{\left(\frac{1}{2\pi fC}\right)}^{2}}≅R.$$ (3)

In our study, $\sqrt{R^{2}+{\left(\frac{1}{2\pi fC}\right)}^{2}}≅R+\left|\frac{1}{j2\pi fC}\right|=R+\frac{1}{2\pi fC}$ since ${\left(\frac{1}{2\pi fC}\right)}^{2}$ or (*R*)^2^ is larger than $\frac{R}{\pi fC}$. The term $\frac{1}{2\pi fC}$ can be eliminated since *R* is larger than $\frac{1}{2\pi fC}$ while *f* is a high frequency. Finally, the magnitude of a series *RC* circuit is simplified as that of a resistor circuit *R*.

The magnitude of a parallel RC circuit is derived as

$$\left|R//\frac{1}{sC}\right|=\frac{1}{\sqrt{R^{2}+{\left(\frac{1}{2\pi fC}\right)}^{2}}}\approx 0.$$ (4)

The term $\frac{1}{R}$ can be eliminated since ${\left(2\pi fC\right)}^{2}>>{\left(\frac{1}{R}\right)}^{2}$ when *f* is a high frequency. The magnitude is simplified as that of a capacitor circuit $\frac{1}{2\pi fC}$. If the signal frequency *f* increases, $\frac{1}{2\pi fC}$ approaches zero and the circuit can be regarded as a short circuit.

This work develops a method for estimating the impedances of a body based on the above two simplified procedures with series and parallel *RC* circuits in various signal frequency bands.

#### Grounded high-pass system for evaluating Re, Rm, Rb, Cm, Cb, Ct, and CX

**Fig 3:**
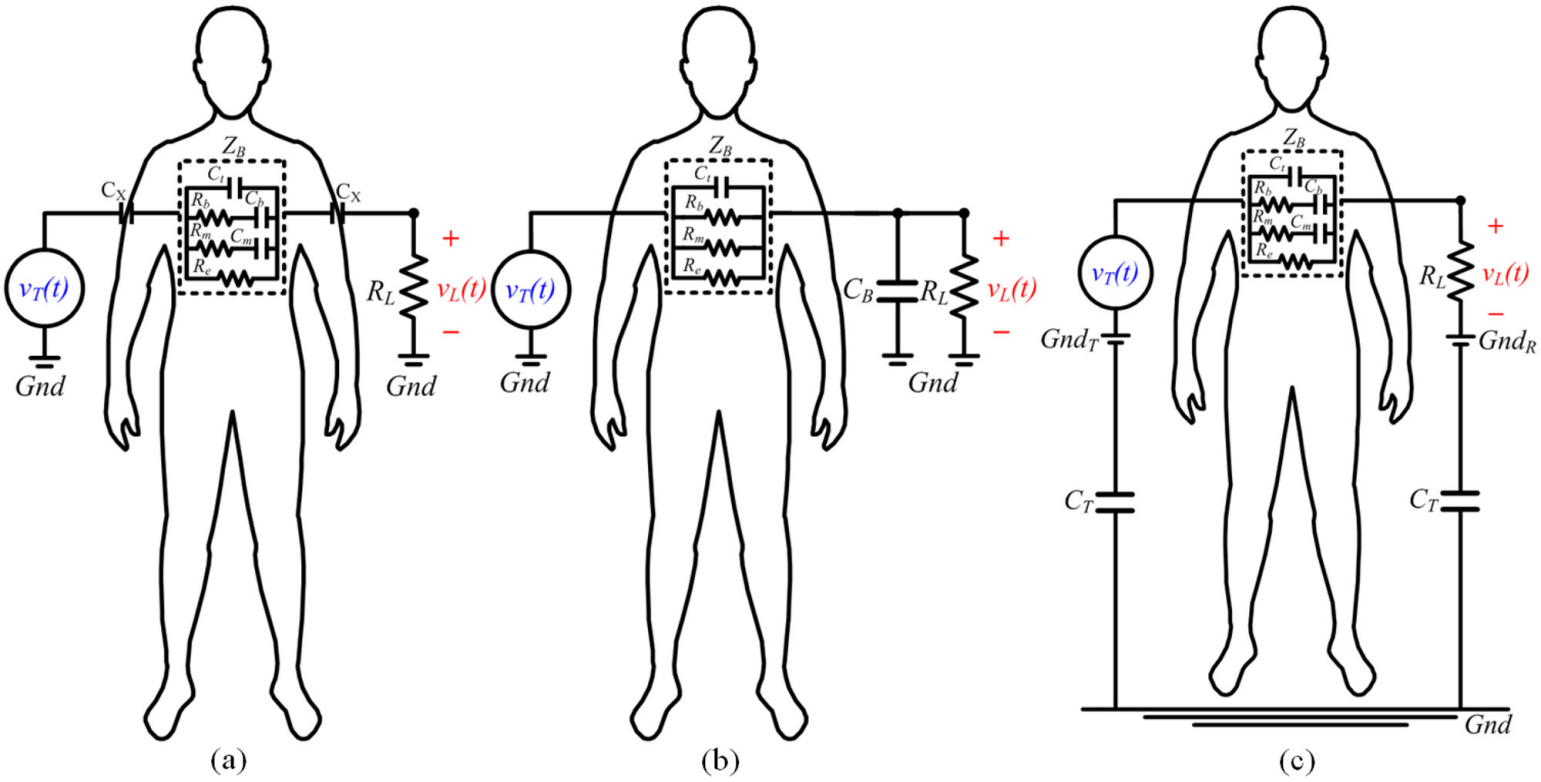
(a) Grounded high-pass system; (b) grounded low-pass system; (c) ungrounded high-pass system.

A high-pass system transfer function is constructed from the characteristic attributes of parallel combinations of *R*_*X*_ and *C*_*X*_, and *R*_*L*_ and *C*_*B*_. Dry skin resistance *R*_*X*_, and dry skin capacitance *C*_*X*_, are typically over several hundreds of kΩ and several tens of *n*F, respectively. As mentioned above, *R*_*X*_ can be generally regarded as open and neglected in an *RC* parallel circuit because *R*_*X*_ is several hundred times larger than the reactance of the capacitance in parallel *C*_*X*_. An *R*_*L*_ value and a specific frequency range are selected, such that $\left|\frac{1}{sC_{B}}\right|>>R_{L}$ and $R_{X}>>\left|\frac{1}{sC_{X}}\right|$. Hence, *C*_*B*_ and *R*_*X*_ are eliminated from Eq 1. Fig 2 can be simplified as a high-pass system (Fig 3a) and Eq 1 is simplified as

$$\left|H(s)\right|≅\frac{R_{L}}{R_{L}+Z_{B}+\left|\frac{1}{sC_{X}}\right|}\ldots .Z_{B}=R_{e}//(R_{m}+\left|\frac{1}{sC_{m}}\right|)//(R_{b}+\left|\frac{1}{sC_{b}}\right|)//\left|\frac{1}{sC_{t}}\right|.$$ (5)

Selecting a frequency band such that $R_{e}<<\left(R_{m}+\left|\frac{1}{sC_{m}}\right|\right)<<\left(R_{b}+\left|\frac{1}{sC_{b}}\right|\right)<<\left|\frac{1}{sC_{t}}\right|$, eliminating the term $\left(R_{m}+\left|\frac{1}{sC_{m}}\right|\right)$*)*, $\left(R_{b}+\left|\frac{1}{sC_{b}}\right|\right)$ and $\left|\frac{1}{sC_{t}}\right|$ from *Z*_*B*_. Eq 5 then becomes

$$\left|H(s)\right|≅\frac{R_{L}}{R_{L}+R_{e}+\left|\frac{1}{sC_{X}}\right|}.$$ (6)

where *R*_*e*_ and *C*_*X*_ are estimated from Eq 6 by applying the piecewise-linear interpolation method to *|H(s)|* versus the corresponding frequency. A frequency band is then chosen to eliminate *C*_*b*_, *R*_*b*_, and *C*_*t*_, and Eq 6 becomes

$$\left|H(s)\right|≅\frac{R_{L}}{R_{L}+R_{e}//(R_{m}+\left|\frac{1}{sC_{m}}\right|)+\left|\frac{1}{sC_{X}}\right|}.$$ (7)

When *R*_*e*_ and *C*_*X*_ are known, *R*_*m*_ and *C*_*m*_ are obtained from Eq 7. Next, by selecting a specific frequency band such that $\left|\frac{1}{sC_{b}}\right|>>R_{b}$, *R*_*b*_ can be eliminated. Eq 7 is shown as

$$\left|H(s)\right|≅\frac{R_{L}}{R_{L}+R_{e}//(R_{m}+\left|\frac{1}{sC_{m}}\right|)//\left|\frac{1}{s(C_{b}+C_{t})}\right|+\left|\frac{1}{sC_{X}}\right|}.$$ (8)

Using the interpolation method as mentioned, *C*_*b*_+*C*_*t*_ can be acquired. Based on Eq 5, increasing the frequency band to several tens of MHz eliminates *R*_*b*_ in Eq 5 because 2π*fR*_*b*_*C*_*b*_ >> 1. Then *C*_*b*_ and *C*_*t*_ are calculated by Eqs 5 and 8, respectively. Finally, by selecting a reasonable frequency band and by knowing |*H(s)*|, *R*_*e*_, *C*_*X*_, *R*_*m*_, *C*_*m*_, *C*_*b*_, and *C*_*t*_, *R*_*b*_ is derived from Eq 5.

#### Determining CB using a grounded low-pass system

By increasing load resistance *R*_*L*_, the high-pass 3dB frequency *f*_*h*_, in Eqs 1 and 2 are allowed to be close to zero and a specific frequency range is selected such that $\left|\frac{1}{sC_{X}}\right|≅0$, $\left|\frac{1}{sC_{m}}\right|≅0$ and $\left|\frac{1}{sC_{b}}\right|≅0$. Thus, the *C*_*X*_ and *C*_*m*_ are shorted. The system becomes a low-pass system. Fig 2 can be simplified as Fig 3b. Eq 1 becomes

$$\left|H(s)\right|≅\frac{R_{L}//\left|\frac{1}{sC_{B}}\right|}{\left(R_{e}//R_{m}//R_{b}//\left|\frac{1}{sC_{t}}\right|)+(R_{L}//\left|\frac{1}{sC_{B}}\right|\right)}.$$ (9)

When *|H(s)|*, *R*_*L*_, *R*_*e*_, *R*_*m*_ and *R*_*b*_ are known, *C*_*B*_ is derived by Eq 9.

#### A ground-free high-pass system for determining CT

Both the transmitted signal, *v*_*T*_(*t*), and the received signal, *v*_*L*_(*t*), are ungrounded (Fig 3c). By decreasing the *R*_*L*_ and increasing the signal frequency within a specific range, the system transfer function becomes a high-pass system:

$$\left|H(s)\right|≅\frac{R_{L}}{Z_{B}+R_{L}+2\times \left|\frac{1}{sC_{T}}\right|}.$$ (10)

With knowing the signal frequency, *Z*_*B*_, *R*_*L*_, and *|H(s)|*, the *C*_*T*_ can be derived using Eq 10.

#### Analytical results

**Fig 4:**
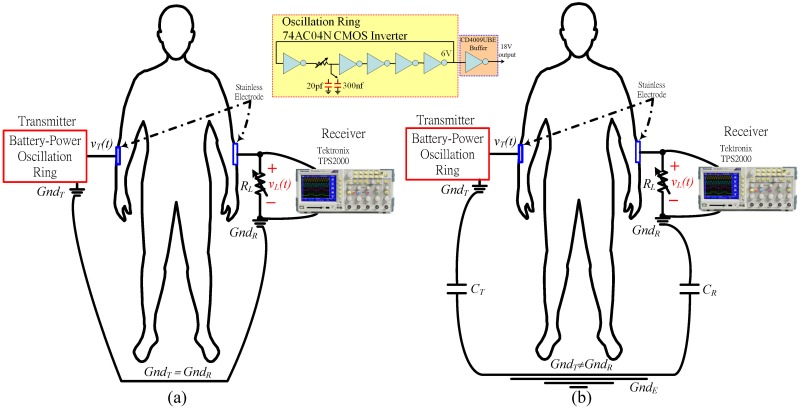
Diagram of the experiment setup. (a) The measurement of the grounded high- and low- pass system, and (b) of an ungrounded high-pass system.

The worst-case scenario of an ESC IBC system exists between the right and left wrists [4–6]. Stainless steel electrodes with an area of 6 cm^2^ are used in the measurement procedure herein, which includes the estimation of body impedance and the analysis of the ESC IBC channel. The electrodes connected directly to the measured body without using gel reduce the skin-electrode impedance. Fig 4 displays the experiment setup for estimating body impedance. Fig 4a presents the measurement of the grounded high- and low- pass system in Fig 3a and 3b, while Fig 4b shows the measurement of an ungrounded high-pass system, which is displayed in Fig 3c.

This experiment places a battery-powered ring oscillator made by a 74AC04N inverter and a CD4009UBE buffer on the left wrist. A battery-powered Tektronix TPS2000 oscilloscope connects to the right wrist. Stainless steel electrodes without gel are connected the body measurement sides and directly to the ring oscillator and oscilloscope. The ring oscillator produces 0−6V and 0−18V square wave with a 52% duty cycle for grounded and ungrounded measurements, respectively, and are induced into the measured body. Parameters are evaluated *via* the following steps.

1. Stimulus signals, *v*_*T*_*(t)*, and body output signals, *v*_*L*_*(t)*, are measured from the output of the square wave generator and the measured human body, respectively.
2. The measured *v*_*T*_*(t)* and *v*_*L*_*(t)* are transformed into the frequency domains *V*_*T*_*(s)* and *V*_*L*_*(s)* using the Matlab tool.
3. Dividing *V*_*L*_*(s)* by *V*_*T*_*(s)* yields *|H(S)|*. Based on Eqs 5–9, the average body parameters are derived by interpolating *|H(S)| versus* a specific frequency.

**Fig 5:**
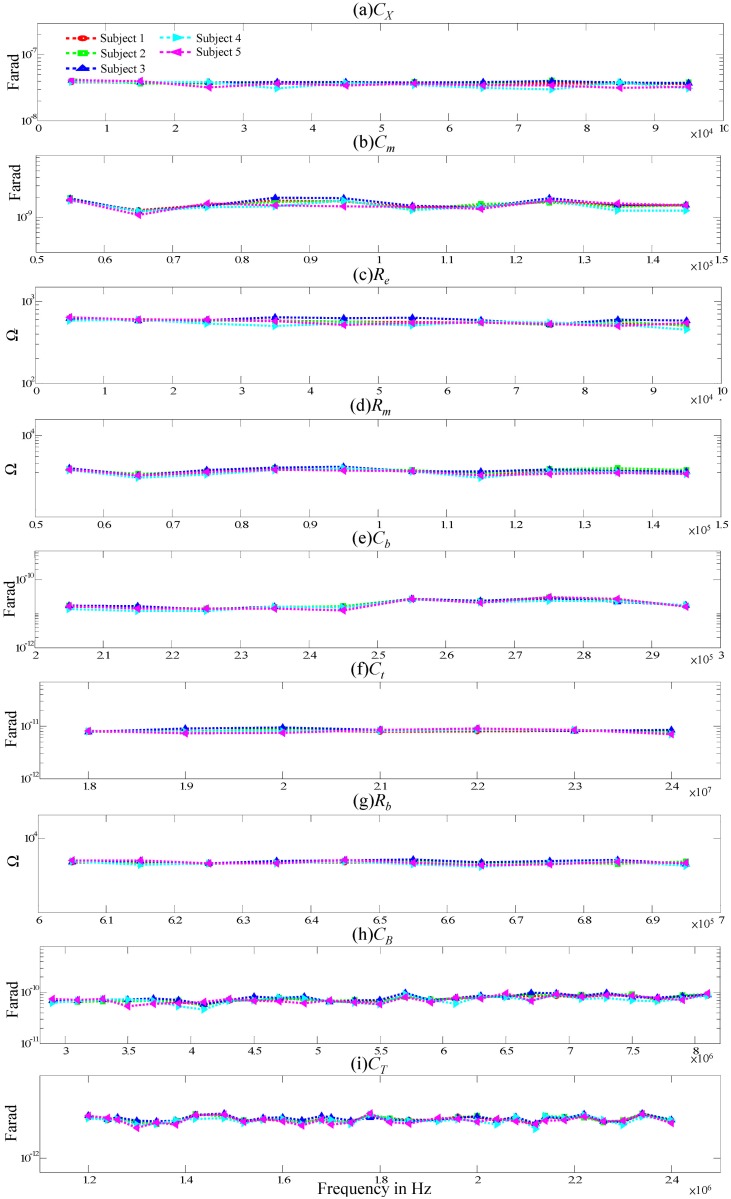
The evaluated body impedances of the subjects.

The five male subjects were evaluated; their ages ranged from 24 to 45 years old, their heights ranged from 1.64 to 1.82m, and their weight ranged from 66 to 82 kg. Fig 5 shows the evaluated body impedances of the subjects. Due to circuit simplification and noise effect on the measured body, the evaluated body impedances vary slightly and approximate to a constant in an appropriate frequency range which is same as the description in above procedures about high- and low-pass system translation. Table 2 summarizes the evaluation and average values for the body impedance and corresponding measurement parameters. Notably, *Z*_*B*_ is 594−414Ω, which corresponds to a signal frequency of 500k−40MHz.

**Table 2:** Evaluation results of the body impedance in Fig 2 and corresponding measurement parameters.

Parameters	*R*_*L*_ (Ω)	Specific frequency band	Square wave frequency	Left Wrist To Right Wrist
*C*_*X*_	25	5k Hz	5k Hz	39.5 nF
*R*_*e*_	25	5k Hz	5k Hz	774
*C*_*m*_	25	55k ~ 145k Hz	5k Hz	1.4 nF
*R*_*m*_	25	55k ~ 145k Hz	5k Hz	2.73k
*C*_*b*_	25	205k ~ 295k Hz, 18M ~ 24M Hz	5k Hz, 2M Hz	17.3 pF
*C*_*t*_	25	205k ~ 295k Hz, 18M ~ 24M Hz	5k Hz, 2M Hz	2 pF
*R*_*b*_	25	605k ~ 695k Hz	5k Hz	3.6k
*C*_*B*_	10k	3M ~ 7M Hz	100k Hz	108 pF
*C*_*T*_	25	1.2M ~ 2M Hz	20k Hz	8.5 pF

### Analysis of the channel characteristics of the baseband signal

**Fig 6:**
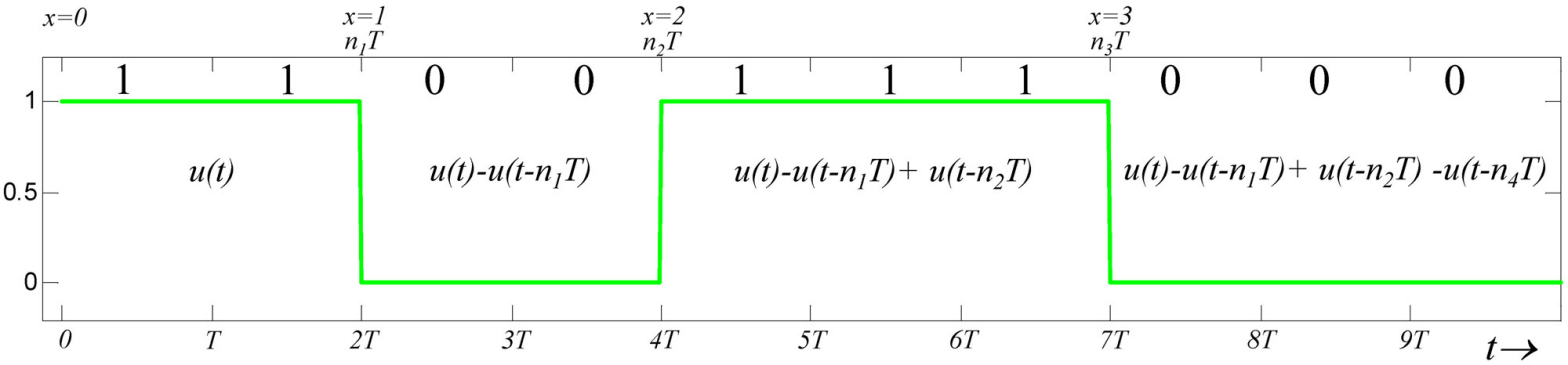
A random digital signal expressed using Eq 11.

The equivalent random digital signal, *v(t)*, with a duration *T* has a common form expressed using the unit step function;

$$v(t)=\overset{\infty }{\underset{x=0}{\sum }}{\left(-1\right)}^{x}u(t-n_{x}T).$$ (11)

where, *x* is the number of data transitioned from 1 to 0 or 0 to 1; and *n*_*x*_ is the number of bits of transmitted data at the *x*^*th*^ transition. Here, *n*_0_ = 0 and *n*_*x*_ > *n*_x−1_; and *n*_*x*_ − *n*_x−1_ determines the number of 1s or 0s being transmitted repeatedly in a run with a probability of ${\left(\frac{1}{2}\right)}^{n_{x}-n_{x-1}}$. Fig 6 shows a random digital signal expressed by Eq 11. The signal with amplitude *A* is transmitted over the channel. The received signal *v*_*L*_(*t*) can be derived as

$$v_{L}(t)=G\times \overset{\infty }{\underset{x=0}{\sum }}\left[{\left(-1\right)}^{x}\times u(t-n_{x}T)\times \left(e^{-2\pi f_{h}(t-n_{x}T)}-e^{-2\pi f_{l}(t-n_{x}T)}\right)\right],$$

$$G=\frac{A}{Z_{B}C_{B}2\omega_{n}\zeta \sqrt{1-\frac{1}{\zeta^{2}}}}.$$ (12)

**Fig 7:**
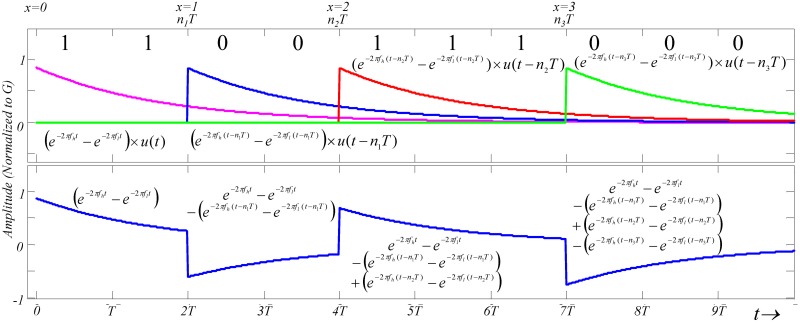
Diagram of a random digital signal transmitted through a band-limitted channel Eq 12.

Fig 7 illustrates a random digital signal transmitted through the channel using Eq 12. For the intersymbol interference (*ISI*) during a transition of the *(x-1)*^*th*^ and *x*^*th*^ data, the received signal *v*_*L*_*(t)* can be expressed as $\left[e^{-2\pi f_{h}t}-e^{-2\pi f_{l}t}\right]-\ldots \ldots \ldots \ldots \ldots \left[e^{-2\pi f_{h}(t-n_{x-1}T)}-e^{-2\pi f_{l}(t-n_{x-1}T)}\right]$. Eq 12 can be reduced as

$$v_{L}(t)=G\overset{x-1}{\underset{m=0}{\sum }}{\left(-1\right)}^{m}\left[e^{-2\pi f_{h}(t-n_{m}T)}-e^{-2\pi f_{l}(t-n_{m}T)}\right].\begin{array}{cc} & n_{x-1}T<t\leq n_{x}T \end{array}.$$ (13)

By observing Fig 7 and Eq 13, the minimum amplitude of *v*_*L*_(*t*) occurs under data transition at *t* = *n*_*x*_*T*. Then, the noise margin, *v*_*nm*_, is derived as

$$v_{nm}(n_{x}T)=G\overset{x-1}{\underset{m=0}{\sum }}\left[{\left(-1\right)}^{m}(e^{-2\pi f_{h}(n_{x}-n_{m})T}-e^{-2\pi f_{l}(n_{x}-n_{m})T})\right].$$ (14)

**Fig 8:**
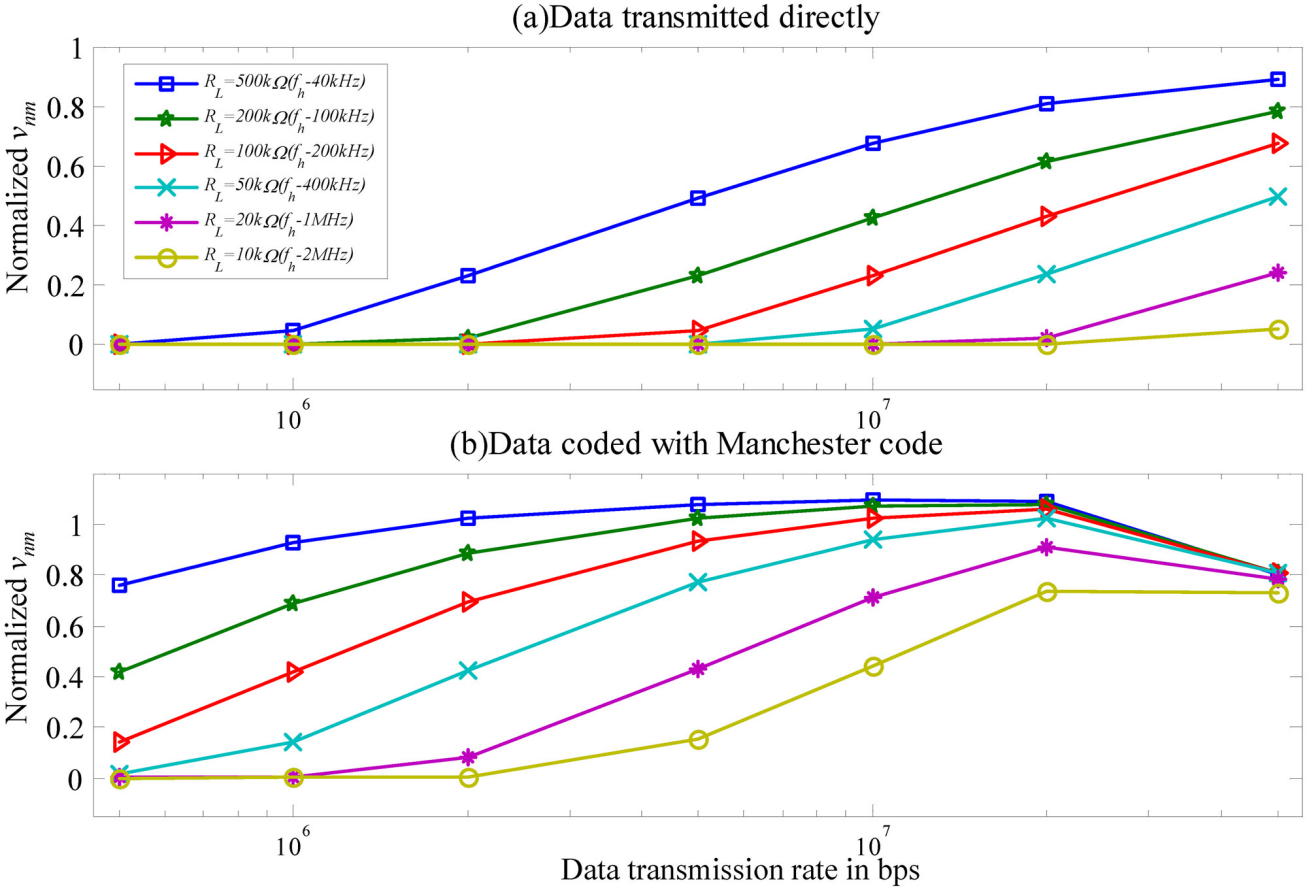
Normalized *v*_*nm*_ *versus* different data transmission rate in the range of 500k−60M bps at the various *f*_*h*_ values between 1k and 1M Hz.

The worst-case scenario of the channel is simulated using the Matlab tool. Fig 8 shows normalized *v*_*nm*_ (12) as function of data transmission rates in the range of 500k−50M bps and *R*_*L*_ in the range of 10k−500k Ω. The corresponding *f*_*h*_ is 1M−40k Hz. This study identifies the effect of the channel on two different baseband signals, including data uncoded and coded with the Manchester code.

A large portion of the signal is filtered by the channel when the data transmission frequency is ≤ *f*_*h*_ and ≥ *f*_*l*_. Data coded with the Manchester code has a larger noise margin than the uncoded data. This improvement is due to the fact that the data coded with the Manchester code shift the low−frequency signal into the channel bandwidth to retain energy.

For a square waveform with a duty cycle of 50%, *n*_*x*_ = *m* and (*m* − 1)*T* < *t* ≤ *mT*. The square wave *v*_L_Sq_(*t*) at the load resistor is obtained by translating in time from Eq 13 and shown as

$$v_{L\_Sq}(t)=G\overset{x-1}{\underset{m=0}{\sum }}{\left(-1\right)}^{m}\left[e^{-2\pi f_{h}(t+mT)}-e^{-2\pi f_{l}(t+mT)}\right],\begin{array}{ccc} & x\to \infty , & 0<t\leq T. \end{array}$$ (15)

The first and second terms of Eq 15 are geometric series with the common ratios of $e^{-2\pi f_{h}T}$ and $e^{-2\pi f_{l}T}$, respectively. A closed form of Eq 15 is derived as

$$\begin{array}{ll} v_{L\_Sq}(t) & =G\left[e^{-2\pi f_{h}t}\left(\frac{1+e^{-2\pi f_{h}xT}}{1+e^{-2\pi f_{h}T}}\right)-e^{-2\pi f_{l}t}\left(\frac{1+e^{-2\pi f_{l}xT}}{1+e^{-2\pi f_{l}T}}\right)\right],\begin{array}{cc} & x\to \infty \end{array},\\ & ≅G\left(\frac{e^{-2\pi f_{h}t}}{1+e^{-2\pi f_{h}T}}-\frac{e^{-2\pi f_{l}t}}{1+e^{-2\pi f_{l}T}}\right),\begin{array}{ccc} & f_{h}\&f_{l}\neq 0, & 0<t\leq T. \end{array} \end{array}$$ (16)

For random data, the probability of datum 1 transmitted repeatedly *n* times in the channel is ${\left(\frac{1}{2}\right)}^{n}$. When number of *n* bit 1 are transmitted repeatedly, the mean amplitude ${\tilde{v}}_{LR}$ is approximated as

$$\begin{array}{l} {\tilde{v}}_{LR}=\frac{1}{T}\left(\frac{1}{2}\overset{T}{\underset{0}{\int }}v_{L\_Sq}(t)dt+{\frac{1}{2}}^{2}\overset{2T}{\underset{T}{\int }}v_{L\_Sq}(t)dt+\ldots \ldots +{\frac{1}{2}}^{n}\overset{nT}{\underset{(n-1)T}{\int }}v_{L\_Sq}(t)dt\right)\\ =\frac{1}{T}\overset{\infty }{\underset{n=1}{\sum }}{\left(\frac{1}{2}\right)}^{n}\overset{nT}{\underset{(n-1)T}{\int }}v_{L\_Sq}(t)dt \end{array}.$$ (17)

A random datum after coding with the Manchester code, the duration of coded data is only *T* and 2*T*. Both probabilities of *T* and 2*T* occurring are $\frac{1}{2}$. Then, mean amplitude ${\tilde{v}}_{LM}$ of the data can be derived as

$${\tilde{v}}_{LM}=\frac{1}{2}\left(\frac{1}{T}\overset{T}{\underset{0}{\int }}v_{L\_Sq}(t)dt+\frac{1}{2T}\overset{2T}{\underset{0}{\int }}v_{L\_Sq}(t)dt\right).$$ (18)

**Fig 9:**
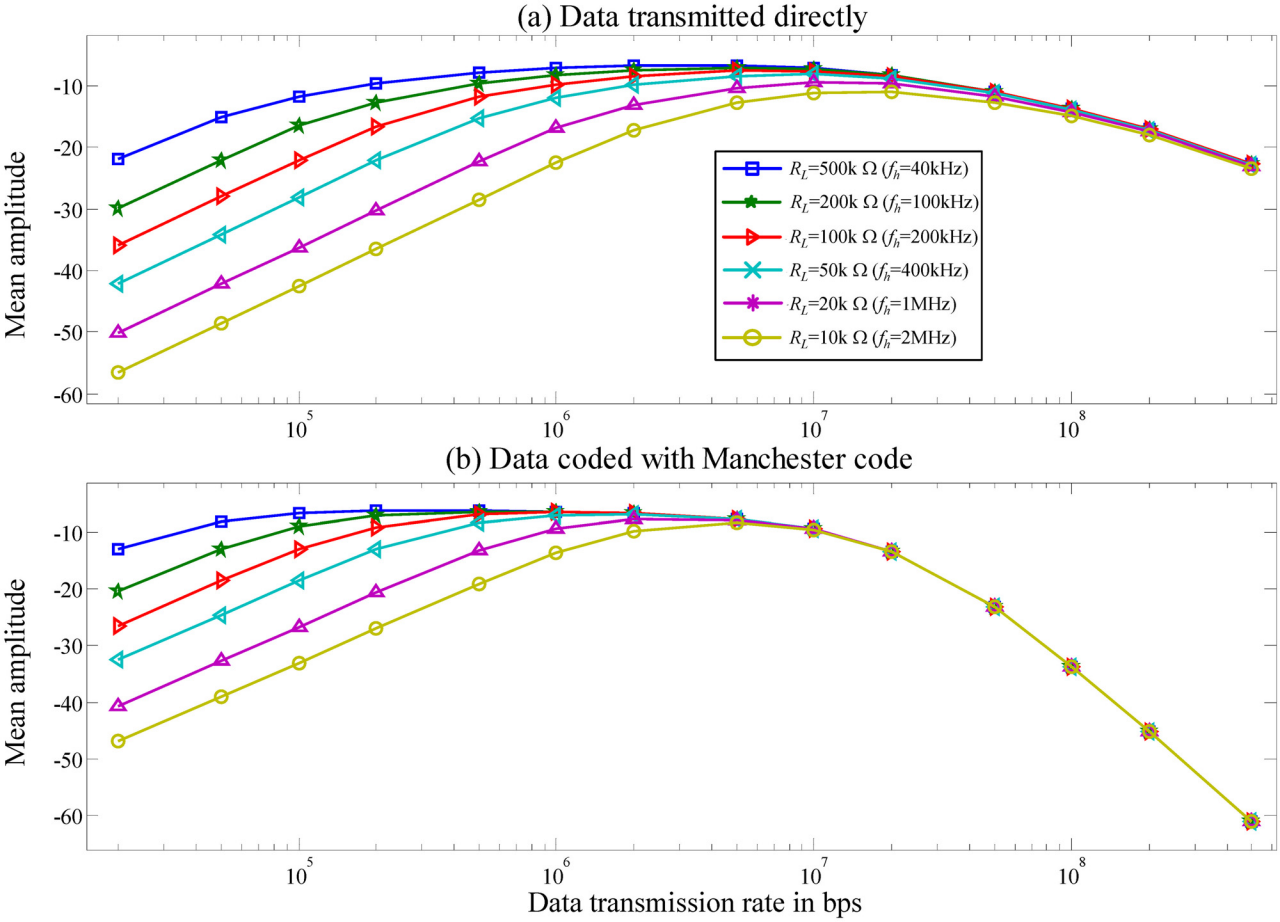
Diagram of mean amplitude calculated from Eqs (17) and (18) *versus* different data transmission rate.

Fig 9 plots the mean amplitude of the coded and uncoded data using different data transmission rate through the channel based on Eqs 17 and 18, respectively. The mean amplitude of the uncoded data is larger than the coded data at a high transmission rate ≥ 20M bps. However, increasing the data transmission rate above *f*_*l*_ increases the deleterious effect of *ISI* on the signal and reduces the noise margin of the signal Eq 14.

Eq 13 indicates that a significant cause of the *ISI* is *f*_*h*_, which determines the tail shape of the transmitted waveform (Fig 7). Moreover, Fig 9, Eqs 17 and 18 show that *f*_*h*_ weakens the energy of the signal at low data transmission rate, especially when data 1 or 0 are transmitted repeatedly for a long period before a transition from 1 to 0 or 0 to 1 occurred. Ultimately, it reduces the noise margin and increases the *ISI*; minimizing *R*_*L*_ increases the *f*_*h*_ and reduces the *ISI*. However, once channel bandwidth is increased, noise increases and the *SNR* decreases proportionally. Hence, system performance is a compromise between the *ISI* and *SNR*. The aim of this work is to achieve the optimal compromise between *ISI* and *SNR* by manipulating *R*_*L*_ to adjust *f*_*h*_.

## Results and Discussion

### Estimated results of the channel SIR

**Fig 10:**
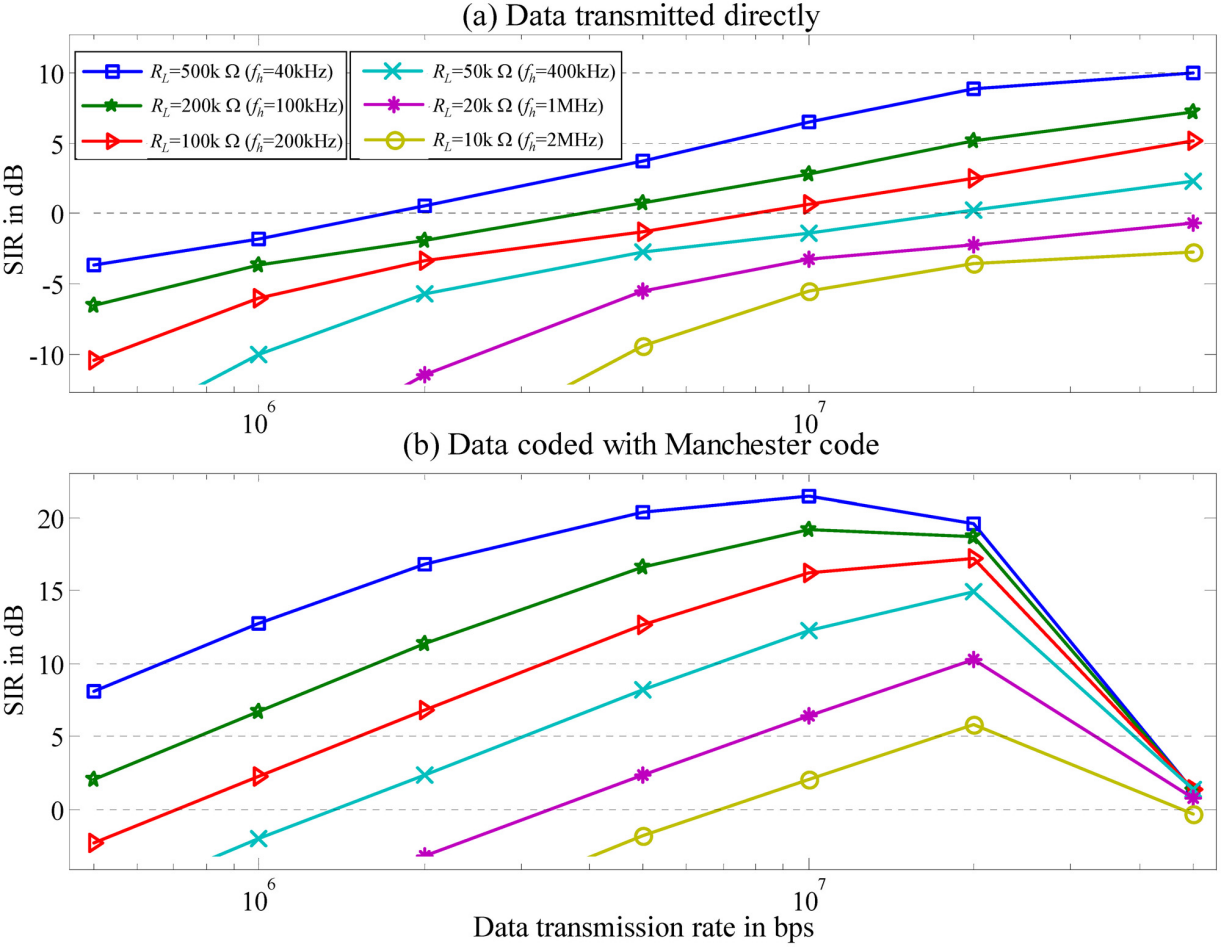
Estimated *SIR*. (a) Data transmitted directly. (b) Data coded with Manchester code.

The channel *SIR* is a function of *f*_*h*_ and *f*_*b*_ (Fig 10). For the data transmission rate *f*_*b*_ slower than 50M bps, the *SIR* reaches its peak. In addition, the *SIR* increases and *f*_*h*_ decreases as *R*_*L*_ increases. Finally, when *f*_*b*_ is much smaller than 50M bps, data coded with the Manchester code has a better *SIR*.

### Estimating channel SNR

**Fig 11:**
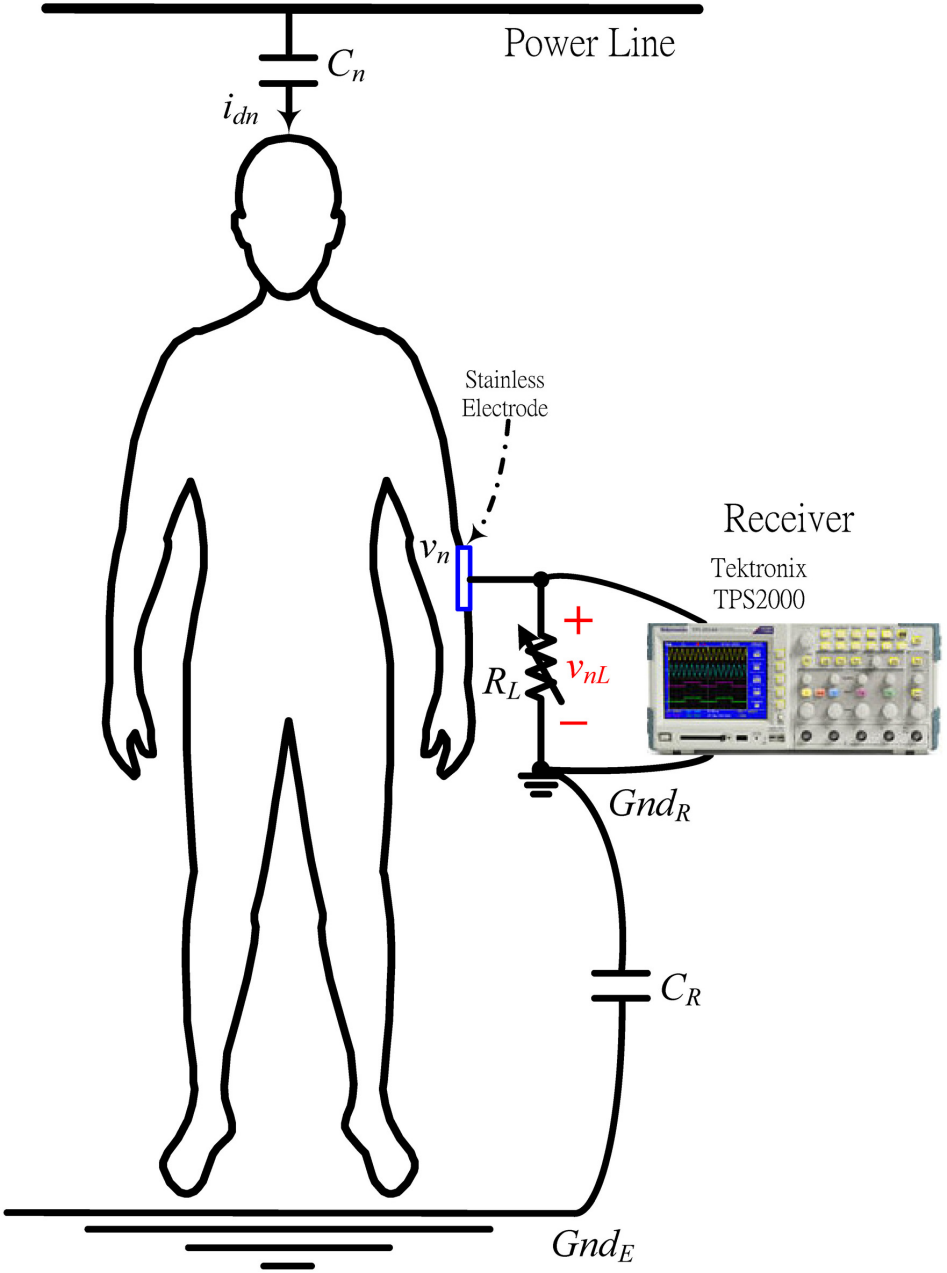
Measurement setup of the channel noise of the ESC IBC system.

Fig 11 shows the setup for measuring 60 Hz power-line noise. A variable resistance *R*_*L*_ from 10k to 1M Ω, provides a corresponding *f*_*h*_ from 2MHz−20kHz. A Tektronix TPS2000 oscilloscope measures body noise from the 60 Hz power line. A noise path from the power line to the human body is modeled as a capacitance *C*_*n*_. A displacement current *i*_*dn*_ flows into the human body from the power line via *C*_*n*_. *v*_*n*_ is the body noise that generated from the displacement current *i*_*dn*_, where *C*_*R*_ is as description in Fig 2. Voltage *v*_*nL*_ represents a noise voltage across *R*_*L*_, and derived as

$$v_{nL}≅\frac{R_{L}}{R_{L}+\left|\frac{1}{sC_{T}}\right|}\times v_{n}.$$ (19)

A 60 Hz noise *v*_*nL*_ with high voltage saturates the front-end amplifier of the receiver. Eq 19 indicates that reducing *R*_*L*_ reduce the noise voltage that is coupled from the power line. A battery-powered device such as a TPS2000 oscilloscope, a transmitter or a receiver in an ESC IBC system can reduce power line noise because the received noise across the load resistor *R*_*L*_ is divided by $R_{L}+\left|\frac{1}{sC_{T}}\right|$.

**Fig 12:**
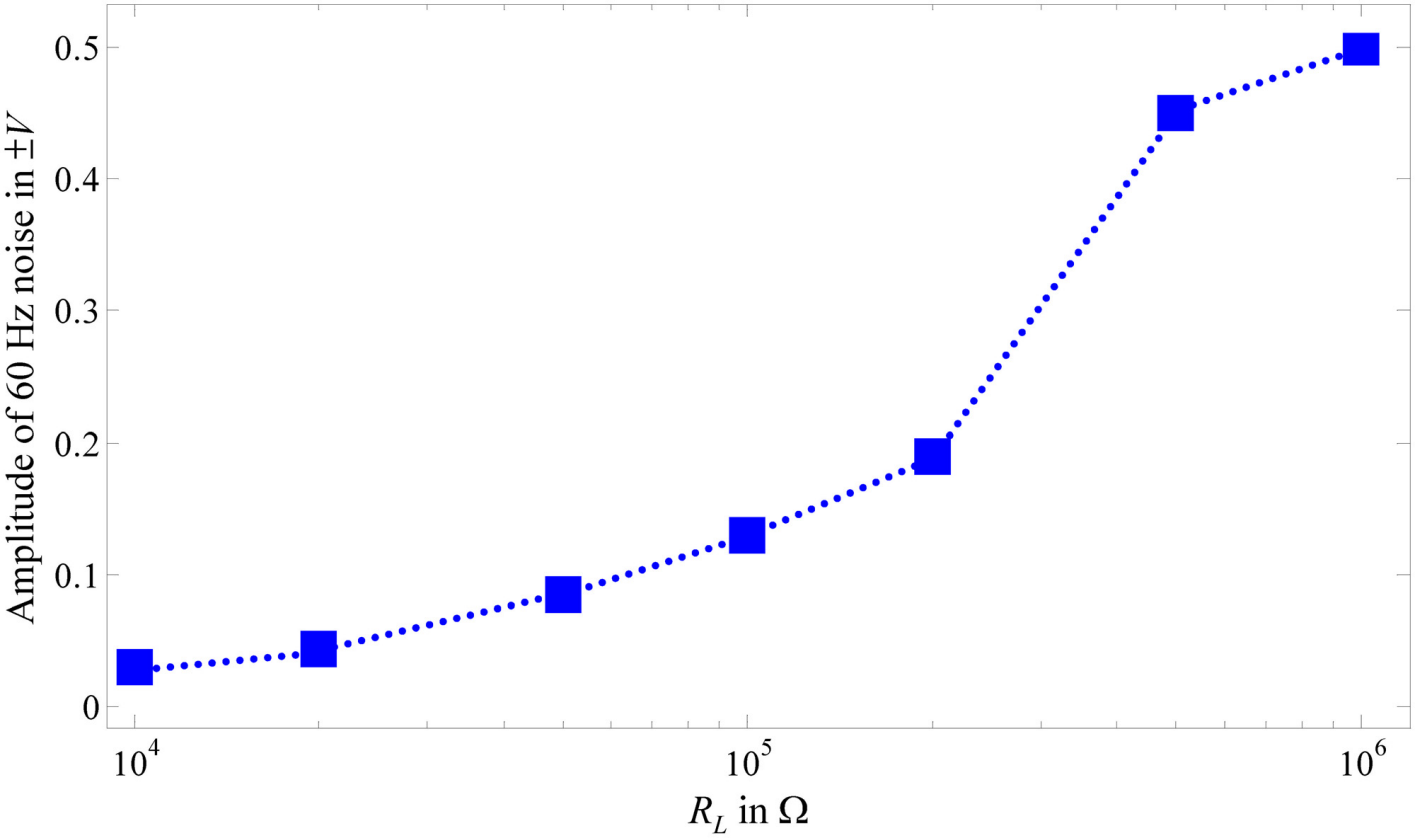
Measurement results of body noise from 60 Hz power line.

Fig 12 plots measurements of mainly 60 Hz power-line noise with amplitudes from ±0.5 to ±0.03 V for different *R*_*L*_ values. The results indicate that the noise channel is a high-pass system, which outputs a noise voltage whose amplitude is directly proportional to *R*_*L*_ and inversely proportional to *f*_*h*_. This finding shows that reducing *R*_*L*_ increases *f*_*h*_ and reduces the 60 Hz power-line noise, consistent with Eq 19.

**Fig 13:**
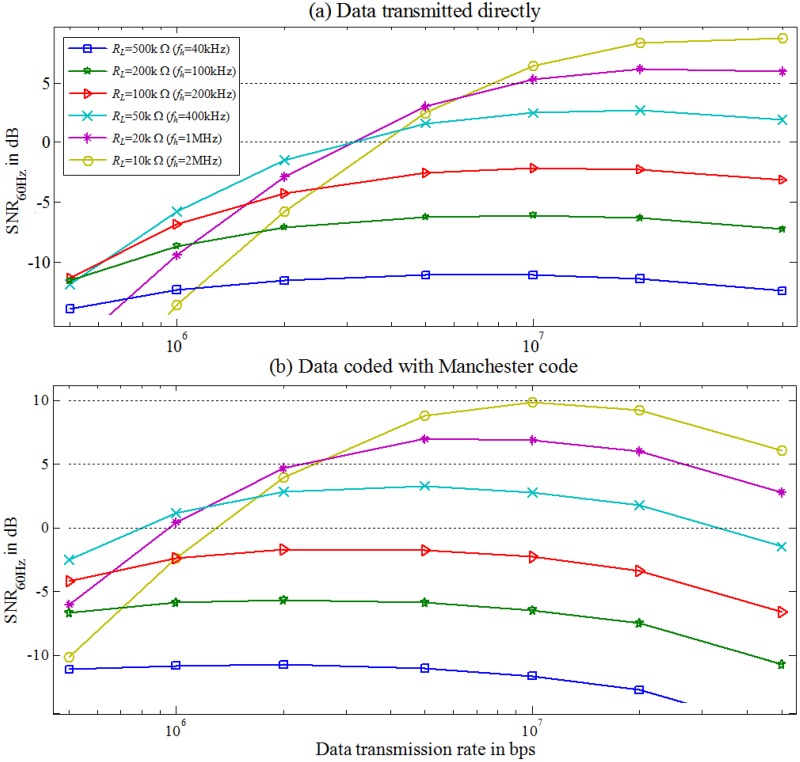
Estimated *SNR*. (a) Data transmitted directly. (b) Data coded with Manchester code.

The *SNR* in the worst-case scenario is estimated based on the estimated *v*_*nm*_ in Eq 14 and measured channel noise (Fig 12). Fig 13 presents the estimated *SNR versus* different *f*_*h*_ and data transmission rate *f*_*b*_ for a baseband signal with an amplitude of 3.3 V transmitted through the channel.

The *SNR* curve shows that increasing *f*_*b*_ increases signal energy in the high-frequency band and decreases it in the low-frequency band. When the data transmission rates are less than 20M bps, data coded with the Manchester code have a higher *SNR* than the uncoded data (Fig 10). At data transmission rate above 20M bps, the coded data has a lower *SNR* than the uncoded data.

By observing Figs 10 and 13 shows that the coding data performs a minimum requirement for both the *SIR* and *SNR* to exceed 3.5 dB when *R*_*L*_ is 50k−10k Ω and the corresponding *f*_*h*_ is 400k−2M Hz. Table 3 summarizes the optimal ranges for *f*_*b*_ for the coding data with a supplied-voltage of 3.3 V in the worst-case scenario to have an *SIR* and *SNR* greater than 3.5 dB.

**Table 3:** Optimum range of the data transmission rate, *f*_*b*_, for signals coded with Manchester code.

*R*_*L*_(Ω)	*f*_*h*_ (Hz)	*f*_*b*_ (bps)	*Estimated SIR (dB)*	*Estimated SNR (dB)*
50k	400k	2M≤ *f*_*b*_ ≤20M	9~14	3.5
20k	1M	5M≤ *f*_*b*_ ≤20M	3.5~10	7
10k	2M	10M≤ *f*_*b*_ ≤20M	3.5~6	9

### Verification

**Fig 14:**
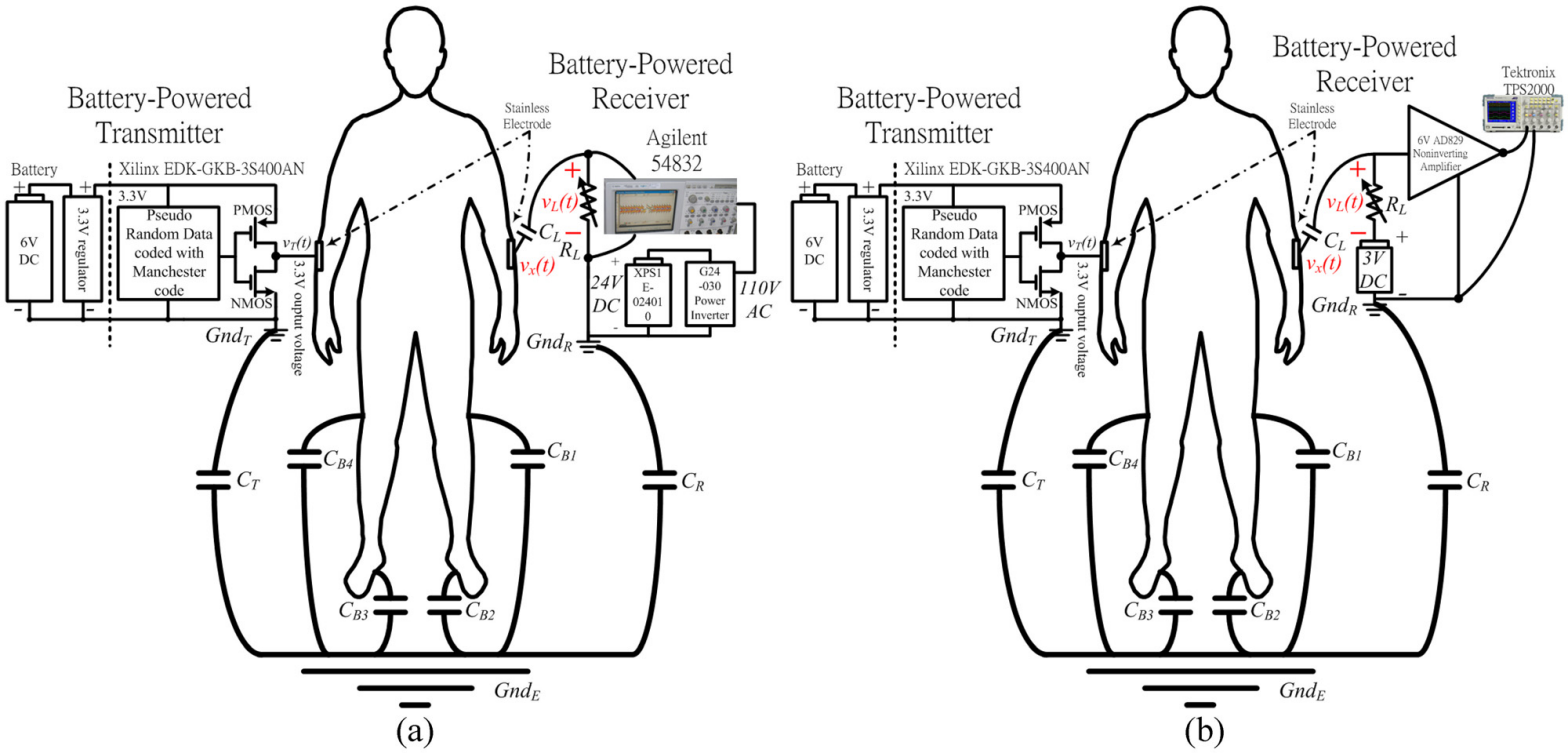
Experimental setup of the measuring (a) eye diagram and (b) typical waveform.

Fig 14 presents the experimental setup that involves a model of the signal return path between the transmitter and the receiver, where *C*_*T*_, *C*_*R*_, and *R*_*L*_ are as in Fig 2; *v*_*L*_*(t)* is the received voltage across *R*_*L*_, and *v*_*x*_*(t)* is the transmitted voltage at the electrode of the receiver. The signal return path is modeled as a series of capacitance, $\frac{C_{T}}{1+\frac{C_{T}}{C_{R}}}$. In the ESC IBC system, the capacitance along the signal return path is $\frac{C_{T}}{2}$ since the areas of the transmitter and the receiver ground are approximately equal so *C*_*T*_ = *C*_*R*_. With respect to the measurements in Fig 14, since the ground areas of the measuring instruments, 54832D and TPS2000 oscilloscope, are larger than the ground area of the transmitter, *C*_*R*_>>*C*_*T*_, so the capacitance along the signal return path is approximately *C*_*T*_.

The measured values of *v*_*L*_ obtained using the proposed measurement system and the ESC IBC system are derived as $\frac{R_{L}}{R_{L}+\left|\frac{1}{sC_{T}}\right|}\times v_{x}$ and $\frac{R_{L}}{R_{L}+\left|\frac{2}{sC_{T}}\right|}\times v_{x}$, respectively. The above equations demonstrate that ESC IBC system with a high pass filter channel has a high pass cutoff frequency $f_{h}=\frac{1}{\pi R_{L}C_{T}}$, which is double that $f_{h}=\frac{1}{2\pi R_{L}C_{T}}$ of the measurement system. Comparing the values of *v*_*L*_ in both the *f*_*h*_, the gain of the measurement system is double that of the ESC IBC system because *R*_*L*_ is eliminated from the denominator in both equations when $\left|\frac{1}{sC_{T}}\right|$ and $\left|\frac{2}{sC_{T}}\right|>>R_{L}$ and the signal frequency is less than *f*_*h*_. When the signal frequency exceeds *f*_*h*_, the received *v*_*L*_ in both systems have same magnitude because $\left|\frac{1}{sC_{T}}\right|$ and $\left|\frac{2}{sC_{T}}\right|$ can be neglected as $\left|\frac{1}{sC_{T}}\right|$ and $\left|\frac{2}{sC_{T}}\right|<<R_{L}$. Hence, the average energy error of the two systems can be estimated as $\left(1-\frac{1-\frac{f_{h}}{f_{l}}}{1-\frac{f_{h}}{2f_{l}}}\right)\times 100\%$. In the proposed system in Fig 2, *f*_*l*_ is 40MHz, *f*_*h*_ is 500kHz, and the estimated error is about 0.63%. The accuracy sufficies for measuring the channel characteristics of the ESC IBC system.

### Measuring the eye diagram of the received data

This work compares the transmission of the coded and uncoded data using an eye diagram that was obtained at *R*_*L*_. The data transmission rate was set between 500kbps and 50Mbps, and *R*_*L*_ = 20kΩ, yielding a *f*_*h*_ ≅ 1M Hz, which is in the optimal range, as stated in the previous section. Fig 14a shows the experimental setup. A battery-powered Xilinx EDK-GKB-3S400AN FPGA is used to transmit a pseudo-random signal that is generated by an m-sequence linear feedback shift register with a length of 23 and an output voltage of 3.3V. Additionally, the baseband digital signal that is transmitted through the human body is measured using an Agilent 54382D oscilloscope with an isolated-battery-powered unit that comprises a 24V LiFe battery (XPS1E-024010) and a DC-to-AC power inverter (G24-030). The power inverter converts a 24V dc voltage into 110V ac voltage, which powers the oscilloscope. The transmitter and the oscilloscope are connected to the right and the left wrists of the human body, respectively, using a stainless steel electrode (of the type described in Section 2–2), replicating an ungrounded environment, as described in Section 2 and presented in Fig 2.

**Fig 15:**
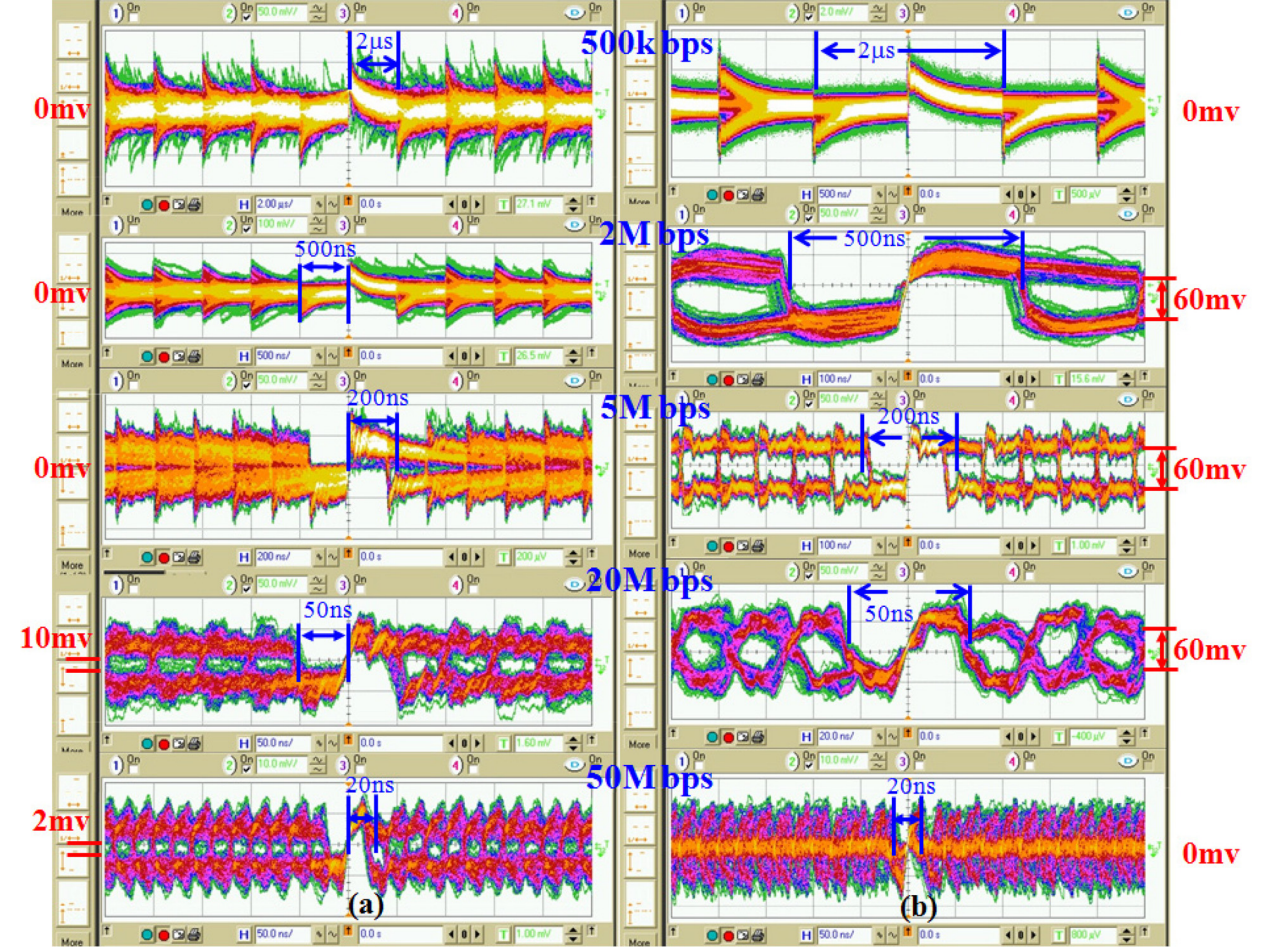
The measured eye diagram of (a) the data transmission directly and (b) the data coded with the Manchester code.

Fig 15 displays the measured eye diagram with data transmission rate of 500k, 2M, 5M, 20M and 50M bps and *R*_*L*_ = 20k Ω (*f*_*h*_ ≅ 1M Hz). Measurement results indicate that the *SNR* increase as the data transmission rate increases for the uncoded data in a data transmission range slower than 50M bps. For the coded data, the *SNR* values of data transmission rates between 2M and 20M bps are larger than those of data transmission rates below 2M bps and above 20M bps. The eye diagram of the coded data includes a 60mV white space, which is 80% of the total area of the eye diagram and a *SNR* of around 10 dB with a data transmission rate of 5M−20M bps. Further, data transmitted directly includes a 10 mV white space, which is 15% of the total area of the eye diagram and has a *SNR* of roughly 2 dB with a data transmission rate of 20M−50M bps.

### Measuring a typical waveform at the output of front-end amplifier

This study measures a typical waveform to verify estimation results (Table 3). Fig 14b presents the experimental setup. The transmitter contains a battery-powered Xilinx EDK-GKB-3S400AN FPGA that generates the pseudo-random signal coded with the Manchester code. The signal period is 2^23^−1; output voltage is 3.3 V; and data transmission rates are 2M, 5M, 10M and 20M bps. The receiver consists of a capacitor *C*_*L*_ (100 nF) for DC isolation, a variable load resistor, *R*_*L*_, and a front-end amplifier. The front-end amplifier is a non-inverting amplifier, an OPAMP AD829, with a gain value of 5 times. The transmitter and receiver are connected to the right and left wrist, respectively, replicating an ungrounded ESC IBC environment. Tektronix TPS2000 battery-powered oscilloscope is used to store the output of the amplifier. The measurement is done with *R*_*L*_ values of 10k, 20k and 50kΩ.

**Fig 16:**
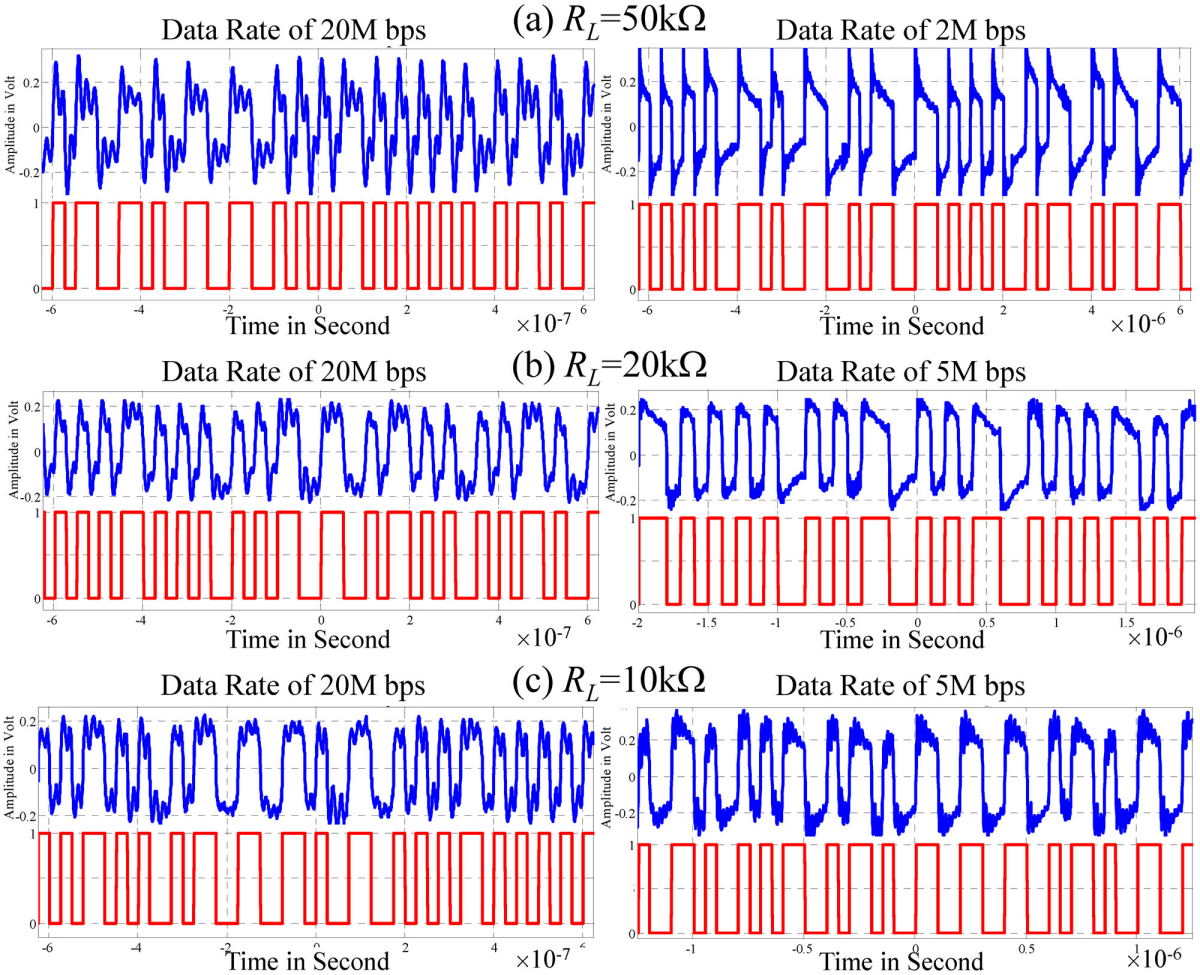
Typical waveforms of the amplifier outputs, (a) *R*_*L*_ = 50kΩ, (b) *R*_*L*_ = 20kΩ and (c) *R*_*L*_ = 10kΩ.

Fig 16 plots the measured amplifier output. Measurement results demonstrate that a capacitor in the channel constructs a signal return path between the transmitter and receiver. The capacitor and *R*_*L*_ form a high-pass response whose 3dB frequency *f*_*h*_ increases as *R*_*L*_ decreases. This experiment verifies that the *ISI* and signal fading are reduced following the increase in the data transmission rate and *R*_*L*_.

The received digital signals (Fig 16) are compared to transmitted digital data without error. The maximum difference in signal width between those signals is less than 2%. The maximum fading of the signal is less than 40% with a system *SNR* exceeding 3.5 dB. Evaluation results are consistent with discussions in previous sections (Figs 10 and 13, Table 3). According to analytical results, a baseband signal coded with the Manchester code with a data rate of 20M bps can be transmitted directly through the ESC IBC channel with good signal quality when *R*_*L*_ in the range of 10k−50k Ω is selected properly.

## Conclusions

This study develops a simplified method to estimate an optima data transmission rate in an ESC IBC system. The method is implemented using an ESC IBC channel model in a ground-free environment. This paper makes the following important contributions. (1) The parameters of the model are evaluated using the developed de-convolution algorithm, which is from a system perspective; (2) a comparative study of two baseband data transmission schemes (with and without Manchester code) in the model in a worst case scenario is conducted using the unit step function, and (3) the optimal baseband data transmission rate for high-speed transmission is obtained by selecting *R*_*L*_ to maximum the *SNR* and *SIR* of the system.

A method of that use battery-powered instruments to imitate the environment of operation of the ESC IBC system. The measurements thus made indicate that the environment provides a signal return path between the ground of the transmitter and that of the receiver in the system. This path can be modeled using a capacitance *C*_*T*_. The *C*_*T*_ and *R*_*L*_ provide a high pass 3 dB cutoff frequency, *f*_*h*_, for the system.

The measurement results also demonstrate that *R*_*L*_ can be simply controlled to achieve an optimal compromise among the *SNR*, *SIR*, and data transmission rate. The optimal range of *R*_*L*_ for Manchester-coded data in the worst-case scenario for ESC IBC channel with a 3.3V supply voltage is estimated at 10kΩ ≤ *R*_*L*_ ≤ 50kΩ, providing a data transmission rate that exceeds 20Mbps, and, therefore, high-speed transmission.

Future work will develop a transceiver (transmitter and receiver) for the ESC IBC system with a high transmission rate of 20Mbps, based on the method proposed herein. The developed transceiver may enhance the ESC IBC system by enabling the integration of multiple electric devices and biosensors that can be used for monitoring human health [44].
